# The Clergy and the Medical Profession

**Published:** 1850-10

**Authors:** 


### EDITORIAL DEPARTMENT.

*The Clergy and the Medical Profession.*—Much has been said, of late, respecting the mutual relations of the clerical and medical professions. Custom and medical ethics prescribe the gratuitous rendering of professional services to the families of those exercising the ministerial office. But in return for this substantial testimony of respect on the part of practitioners of medicine, clergymen are often found among the most active, as well as influential, of the enemies and detractors of medical science. Hence, it is sometimes urged that they should be placed on a par with other patients as respects remuneration for professional attendance. Resolutions to this effect, indeed, have, we believe, been adopted by some local societies. We cannot concur in the propriety of this course as a retaliatory measure. "Two wrongs do not make a right." If the clergy have any valid claims on us for gratuitous services, it does not weaken the force of those claims that the value of our services is not appreciated, or receives an ungrateful return. If physicians were in the habit of declining to serve all except those who placed a proper estimate on the resources of medical art, their practice would be far more exclusive than it now is. The rule of refusing remuneration from clergymen may be a bad one, but, if so, it is for other reasons than that some of the clerical body patronize quacks, give certificates of the value of secret remedies, and officiously engage in electioneering in behalf of homœopathy, Thomsonism, etc. We concede their right to do this if they can reconcile it to their own consciences. We incline to the opinion that it would be better for both professions if the clergy were expected to pay for medical services. Both parties would thereby be more independent, and we are not sure but it would conduce to a higher estimate of medicine, inasmuch as what costs nothing is apt to be deemed worthy of little esteem. We have introduced this topic for the purpose of quoting some remarks thereupon by a clerical writer. We take them from the North-Western Medical and Surgical Journal, and they are credited by that periodical to a religious paper called the Puritan Recorder. We copy the remarks in order to accord a hearing of the clergy in their own defence, and also because, with our esteemed contemporary, we think they contain *sense* as well as *humor*.

"Of late there seem to be symptoms of unhealthy feeling on the part of the medical gentlemen. It is even rumored, that, in some of their Conventions and Societies, they are agitating the project of a breach of the peace with the ministers of the Gospel, because a few of these have soiled their cloth by patronizing and puffing some of the patent nostrums of the day. We have no apology to make for such unministerial conduct, which is highly indiscreet and reprehensible. But this we will say, that, for one respectable and educated clergyman who has stultified himself by being thus mixed up with quackery, at least an hundred have abhorred the contact of 'the unclean thing.'

"Why should the whole body of ministers in regular standing, be made responsible for the errors and follies of a few of their brethren, who, in such matters, are under no ecclesiastical control? Have any of the ecclesiastical bodies, large or small, taken action in the premises? Has the General Association of Massachusetts recommended to the churches the use of Brandreth's Pills? Has the General Assembly of the Presbyterian Church (Old School.) espoused the cause of Sands' Sarsaparilla; or has the New School Assembly taken up the claims of the rival syrup of Townsend? Have the Episcopal bishops given vogue to the big-bellied bottles of Mrs. Kidder's Cordial? Has the General Conference of the Methodist brethren indorsed for the virtues of the Thompsonian hot-drops? Is the American Unitarian Association responsible for Homœopathic doses in medicine as well as in religion? Or has the Baptist communion pledged itself to Hydropathy for any but spiritual purposes? If no such ecclesiastical absurdity has been perpetrated, why is an entire body of unoffending men charged with the extravagances of a few visionary or eccentric brethren, who have ignorantly misapplied their official influence to the support of imposture?

"Here we are bound to warn the gentlemen of the medical profession, that, if they declare war with the ministers on such grounds, they may yet be obliged to drink out of their own measure. We would inquire whether none of them have been known to patronize quack preachers, empirical reformers, and other religious Jack-puddings, who are vending their noxious nostrums in theology and morals, poisoning and stupefying the minds of the people, and spreading the ravages of spiritual death? Is it not a fact, that very many regular-bred physicians haunt the shops of the most notorious theological impostors, and go abroad to bring their pernicious wares into general use? And now what propriety would there be in preaching up a crusade on the part of the clergy against the physicians, as a class essentially radical and faithless?"

In connection with the foregoing, we copy the following article from the Boston Medical and Surgical Journal. It contains an eloquent, and, as we hope, a just tribute to the character of the medical profession, by a member of the clerical body:—

"*The Beloved Physician.*"—A discourse delivered in Norfolk, Conn., by the Rev. Joseph Eldridge, at the funeral of the late Benjamin Welch, M.

D., of the same place, has been received. It seldom falls to our lot to read eulogies pronounced by the clergy upon one of the medical profession, and particularly one that possesses so much character and truthfulness as the one now before us. A just appreciation of the medical profession by the clergy is not common; and when we see that the physician's services are sometimes requited, and that there is one of the clerical profession who has honor and intelligence enough to award him merit, and disseminate just sentiments among the people, we must confess that it is a powerful stimulus to increased efforts in our profession. Some portions of the discourse are so truthful and applicable, that we quote freely from it, hoping that others, besides medical men, will read it, and give it their consideration. In speaking of the duties of physicians, he says, "The services necessarily impose upon him severe exertions, bodily and mental, such as subject him to many privations, and much hardship. He must unavoidably be irregular with respect to food and sleep; he has no command of his time, is subject to every body's call; when summoned, he must go, whether fresh or weary, whether it be night or day, whatever be the state of the roads or the weather. He cannot be excused from rising from his bed to-night, after having just comfortably deposited himself in it, because he entered no bed last night, nor, it may be, the night previous. With the most painful and distressing scenes he must be daily conversant; he must pass large portions of his time in sick rooms, discharging disagreeable offices; must be familiar with wounds and diseases, with the sufferings of the sick, and the mortal agony of the dying. *His services are not requited, and many of them are such that they cannot be.* He is expected to be no respecter of persons; he is every man's servant; he is commanded as readily by those who have no means of compensating him, as by those of the amplest resources. *After much reflection upon the subject, I am settled in the conviction that more gratuitous labor is performed by physicians than by any other class in the community.* It has come to be a sort of common law that they must do it. If they should decline visiting a sick family, on account of its poverty or inability to pay for the service, many would hold up their hands in astonishment and horror, who themselves would not render the slightest assistance in the very same case. Even those who have the ability and intention to pay their other debts, are often content to suffer the honest demands of their medical attendants to run along indefinitely. *The per centage of unrequited labor performed by this profession is very large.* Why should it be so? Their time, their strength, and their skill, are their own. Nevertheless, much of their time, strength, and skill, are given away. Then there is much in their services which cannot be requited, for which money is no adequate remuneration. For the weary miles they travel, for the time spent with the sick, for the sacrifice of sleep and physical comfort, they may perhaps be paid—pecuniary compensation may possibly cancel claims for such services. But what shall we say of their frequent solicitudes for the sick in critical cases; their overwhelming anxiety when precious and valuable lives hang on their decisions? *when, in the struggle with disease, they find their efforts baffled; what shall we say of this wear and tear of sensibility and feeling? Will a few dollars and cents cancel such debts? They are not cancelled—they never can be.*"

